# Cellular retinol binding protein 1 transfection reduces proliferation and AKT-related gene expression in H460 non-small lung cancer cells

**DOI:** 10.1007/s11033-020-05744-5

**Published:** 2020-09-09

**Authors:** Amedeo Ferlosio, Elena Doldo, Sara Agostinelli, Gaetana Costanza, Federica Centofanti, Angelo Sidoni, Augusto Orlandi

**Affiliations:** 1 Anatomic Pathology, Department of Biomedicine and Prevention, Tor Vergata University of Rome, Rome, Italy; 2 Dermapathology laboratory, San Gallicano Institute, Rome, Italy; 3 Department of Experimental Medicine, Section of Anatomic Pathology and Histology, Medical School, University of Perugia, Perugia, Italy; 4 Department of Anatomic Pathology, Tor Vergata Policlinic of Rome, Rome, Italy; 5 Institute of Anatomic Pathology, Dept. of Biomedicine and Prevention, Tor Vergata University of Rome, Via Montpellier, 00133 Rome, Italy

**Keywords:** Non-small cell lung cancer, CRBP-1, AKT pathway, Retinoids

## Abstract

In recent years, new treatments with novel action mechanisms have been explored for advanced non-small cell lung cancer (NSCLC). Retinoids promote cancer cell differentiation and death and their trafficking and action is mediated from specific cytoplasmic and nuclear receptors, respectively. The purpose of this study was to investigate the effect of Cellular retinol binding protein-1 (CRBP-1) transfection in H460 human NSCLC cell line, normally not expressing CRBP-1. H460 cells were transfected by using a vector pTargeT Mammalian expression system carrying the whole sequence of CRBP-1 gene. For proliferation and apoptosis studies, cells were treated with different concentrations of *all-trans* Retinoic Acid (*at*RA) and retinol. AKT-related gene expression was analyzed by using western blot and Signosis array and results analysed by one-way analysis of variance (ANOVA) or by t-student test. CRBP-1^+^ showed reduced proliferation and viability in basal condition and after *at*RA treatment when compared to empty-transfected H460 cells. Reduced proliferation in CRBP-1^+^ H460 cells associated to the down-regulation of pAKT/pERK/pEGFR-related genes. In particular, gene array documented the down-regulation of AKT and Stat-3-related genes, including M-Tor, Akt1, Akt2, Akt3, Foxo1, p27, Jun. Restoration of CRBP-1 expression in H460 cells reduced proliferation and viability in both basal condition and after *at*RA treatment, likely by down-regulating AKT-related gene level. Further studies are needed to better clarify how those CRBP-1-related intracellular pathways contribute to counteract NSCLC progression in order to suggest a potential tool to improve efficacy of retinoid anti lung cancer adjuvant therapy.

## Introduction

Lung cancer is the first cause of neoplastic death worldwide in both men and women population [1]. Non-small cell lung carcinoma (NSCLC) accounts for 80% of all cases. Nevertheless the recent progress in NSCLC therapy with the advent of personalized medicine, only 18% all patients are still alive after 5 years from diagnosis [2]. In lung cancer, Vitamin A (retinol) deficiency has been associated with bronchial metaplasia and increased cancer development [3–5]. Retinoids (retinol derivatives) showed to promote differentiation and cell death of cancer cells in a number of experimental systems, including lung [5–7]. In recent years, new treatments with novel action mechanisms have been explored for advanced lung cancer, including retinoids administration [3, 7, 8]. Biological activity of retinoids, in particular *all-trans* retinoic acid (*at*RA) is normally mediated by specific cytoplasmic and nuclear receptors [8–10]. Cellular retinol binding protein-1 (CRBP-1) is a 15 kDa cytosolic binding protein crucial for uptake and subsequent esterification of retinol, by regulating its bioavailability [8, 11]. CRBP-1 is indispensable for embryonic development and growth of vertebrates. In the lung parenchyma, CRBP-1 expressing cells are observed during development and pre-natal alveolus formation [12]. Defects in CRBP-1 gene expression linked to oncogenic process in breast, prostatic, renal, lung and endometrial cancer [13–15]. Retinoids exert their pleiotropic and transcriptional effects binding nuclear receptors, namely the retinoic acid receptors RARα, β, and γ, and retinoid X receptors RXRα, β, and γ [5, 8, 10, 16]. RARs may form homodimers [17, 18] or heterodimers with other receptors as thyroid hormone receptors, Vitamin D3 receptors, peroxisome proliferator active receptors (PPARs) and several orphan receptors, contributing to starting alternative signalling pathways [19]. For example, RARβ and RXRα, in normal respiratory epithelium, binding PPARγ with other cofactors, ensures cyclin D1 mediated cell cycle inhibition hence favouring apoptosis or differentiation [5, 18]. Down regulation of RARβ combined with AP-1 up-regulation triggers tumour progression and proliferation of NSCLC cells [20]. Concurrently, the inability of RXRα to form heterodimers with PPARγ enables an AP-1/CRB-dependent up regulation of Cox2, resulting in inhibition of apoptosis [16, 21]. The loss of RARβ mRNA expression has been observed in many lung cancer cells line and its expression is contingent on intracellular concentration of retinoids, mediated by CRBP-1 and 2 [20]. Moreover, retinoids can activate several pathway, including AKT/ERK signalling in lung cancer cells through a transcriptional independent-mechanism [3, 22]. Retinol can induce cytokine-dependent activation of Jak2 and subsequently STATs transcription factor, while the exchange from RBP to intracellular CRBP-1 is mediated by Stra6 receptor [23].

We recently documented that high expression of CRBP-1 in lung adenocarcinoma in vivo was associated to a lower overall survival [24]. Napoli et al. proposed that CRBP-1 expression in NSCLC should be considered as marker of RARβ down regulation [8]. In fact, authors suggested that CRBP-1 cytoplasmic accumulation represent a block of nuclear availability of retinoids.

In this preliminary study, we investigated the effect of *at*RA in native and CRBP-1-transfected H460 lung cancer cells with particular reference to the modulation of RAR/RXRs and pAKT/pERK/pEGFR gene signaling. We highlighted that the restoration of CRBP-1 influenced the proliferation and AKT signalling pathway in H460 cell line also in the presence of *at*RA treatment.

## Methods

### Cell transfection

Human non-small cell lung cancer H460 cell line (kindly provided by Dr. Carlo Leonetti, Regina Elena National Cancer Institute, Rome, Italy) maintained in RPMI 1640 (Lonza Bio Pharma AG, Switzerland) and transfected by using a vector pTargeT. Mammalian expression system carrying the whole sequence of CRBP-1 gene (NM_002899) and the gene for the resistance to G418 (Promega, Italy), or the G418-resistance gene alone, as reported [25]. After 20 days, stable transfected clones were collected in G418-containing medium and tested by PCR and western blot. The correct plasmid sequence confirmed by Sanger sequencing. Experimental procedures were repeated by using different transfected clones, which gave similar results (data not shown).

### Cell growth, viability and clonogenic assay

For proliferation studies, native and transfected H460 cells were treated with different concentrations of *at*RA (R 2625; Sigma-Aldrich, St. Louis, USA) and retinol (R7632; Sigma-Aldrich, St. Louis, USA) in 0.1% FBS (Fetal Bovine Serum, Sigma-Aldrich) up to 3 days. For cell viability, 3-(4,5-dimethylthiazol-2-yl)-2,5diphenyl-tetrazolimbromide assay (MTT, Sigma-Aldrich) was performed [26]. For the clonogenic assay, after seeding and overnight serum-starving, cells were maintained with 10% or 0.1% of FBS and treated with 5 µM *at*RA. Colonies arising from survival cells were fixed and stained with 1% methylene blue (Sigma-Aldrich) in 0.1% methanol and their percentages as plating efficiency (PE) calculated [24].

### Gene expression analysis

Gene expression analysis was performed using Signosis array (Signosis, Inc., Santa Clara, CA, USA). Briefly, total RNA was extracted [27, 28], reverse-transcribed into cDNA in the presence of biotin-dUTP and a profile of 24 genes for human AKT and Stat-3 pathway cDNA plate array (Catalog number AP-0161 and AP-0151 respectively; Signosis). Luminescence relative light units (RLUs) was evaluated on a microplate luminometer, according to the manufacturer’ instruction. Real-time PCR for RXR, RARs and cytokeratins was also performed in triplicate using β2-microglobulin, β-actin and glyceraldehyde-3-phosphate dehydrogenase (GAPDH) as housekeeping genes, as previously reported [24].

### Western blot analysis

After isolation, content determination and electrophoresis, proteins were elettroblotted [29] and incubated with a polyclonal rabbit anti-CRBP-1, anti-RXRα, anti-RARα (1:500, Santa Cruz Biotechnology, USA), anti-RARβ, anti-RARγ, anti-cytokeratin-5/6, anti-cytokeratin-10 (1:500, Abcam, Cambridge, UK), anti-phosphorylated-AKT (pAKT Ser^473^), anti-AKT, anti-phosphorylated ERK1/2, anti-phosphorylated EGFR (Thr669) and mouse anti-total tubulin antibody (Sigma-Aldrich), followed from horseradish peroxidase conjugate goat anti-rabbit or anti-mouse IgGs (Pierce, Rockford, USA). Specific complexes were revealed and quantified as reported [29] in three independent experiments. AKT and EGFR activity expressed as phospho/total protein ratio [30].

### Patients and methods

Paraffin blocks of tumors of a small cohort of patients with histologic diagnosis of NSCLC (Santa Maria della Misericordia Hospital of Perugia, Italy) were included. Tumor classification was in accordance with WHO criteria and the most diffuse immunohistochemical panel. For CRBP-1 positive and negative expression, tissue sections were incubated for 1 h with rabbit polyclonal anti-CRBP-1 (1:200; clone FL-135, Santa Cruz Biotechnology, Heidelberg, Germany). Diaminobenzidine was used as final chromogen. CRBP-1 expression was estimated at × 400 magnification by two of the Authors by using the following semi-quantitative grading system: absent and strongly positive expression. This retrospective study was approved by Ethics Committee, waiving patient consent.

### Statistical analysis

Results were analysed as the arithmetical mean ± SD Data were analyzed by one-way analysis of variance (ANOVA) followed from a Bonferroni post hoc test or using the Student *t*-test. The differences were considered statistically significant for *p* values < 0.05. All the statistical analyses performed with SSPS V.20 (Stat Corp, College Station, TX, USA).

## Results

### Restoration of CRBP-1 expression reduces survival and clonogenicity of H460 cells

**Fig. 1:**
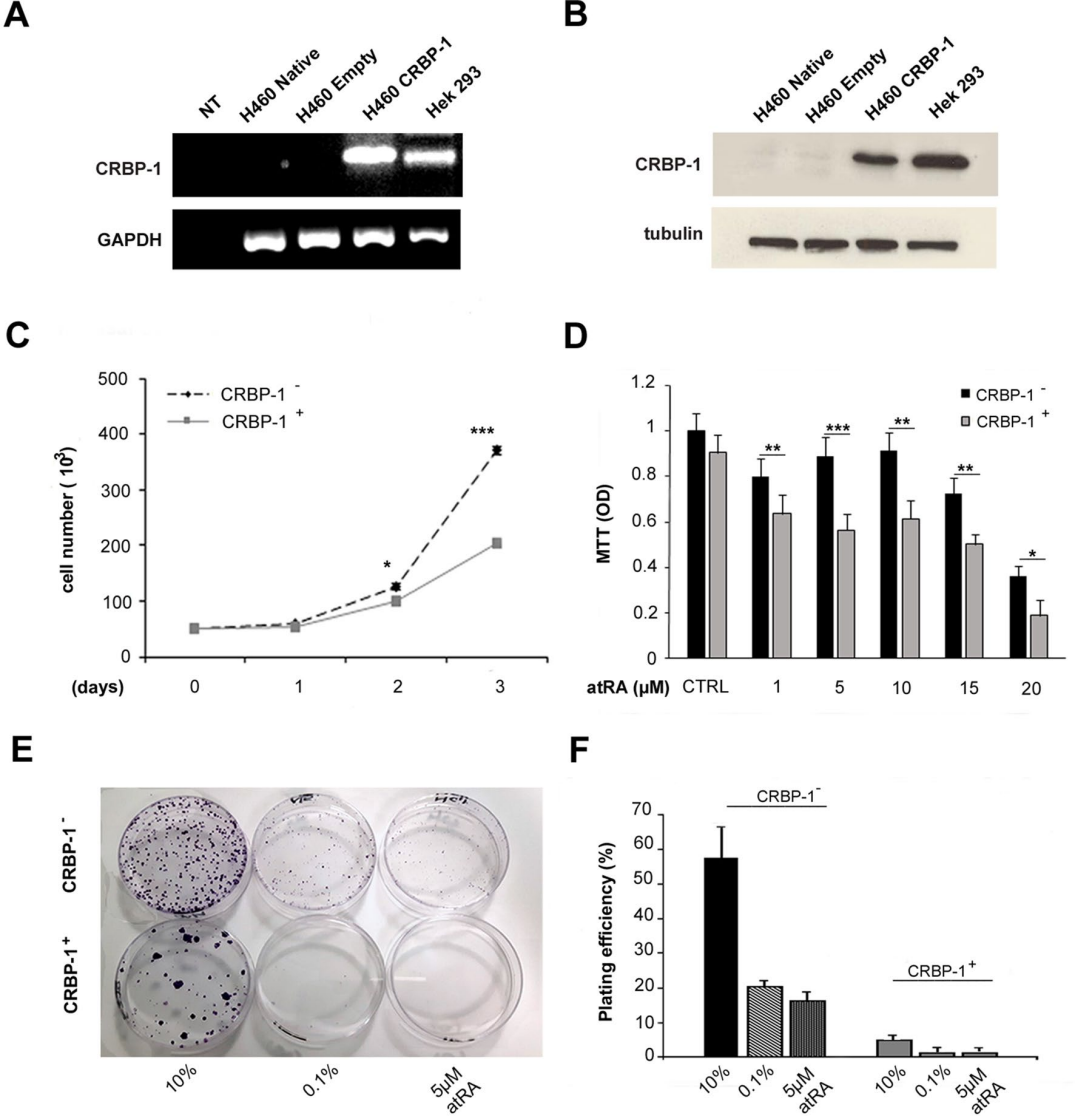
CRBP-1 transfection reduces viability and retinoid-related survival of H460 cells. **a** RT-PCR and **b** representative blots of protein expression in CRBP-1-transfected in H460 cell line. Hek-293 cells as positive control. **c** CRBP-1^+^ H460 cells growth increased compared to empty-transfected cells. **d** MTT assay shows reduced viability of CRBP-1^+^ compared to empty-transfected H460 cells after 2 days of *at*RA treatment in the presence of 0.1% FBS. **e**, **f** CRBP-1^+^ maintained with 10% and 0.1% of FBS and after 48 h of 5 µM *at*RA treatment showed a poor ability to form colonies compared to empty-transfected H460 cells. Values expressed as means ± SD of three different experiments: *p < 0.05, **p < 0.005, ***p < 0.001

As reported in Fig. 1a, b, empty-transfected H460 cells did not express CRBP-1, similarly to native cells. To investigate the effect of CRBP-1 transfection on H460 cell survival, cell counting and MTT assay were performed. Our results showed that 10% FBS-cultured CRBP-1^+^ grew less than empty-transfected H460 cells starting from 2nd day (*p* < 0.05; Fig. 1c). CRBP-1^**+**^ viability was also reduced more markedly in the presence of different *at*RA concentrations (1–20 µM; IC_50_ = 5µM; Fig. 1d) when compared to empty-transfected H460 cells. Finally, CRBP-1^+^ H460 maintained with 10% and 0.1% of FBS and after 48 h of 5 µM *at*RA treatment showed a poor ability to form colonies compared to empty-transfected H460 cells (Fig. 1e, f).

### CRBP-1 transfection influences proliferative and transcriptional gene levels of H460 cells

**Fig. 2:**
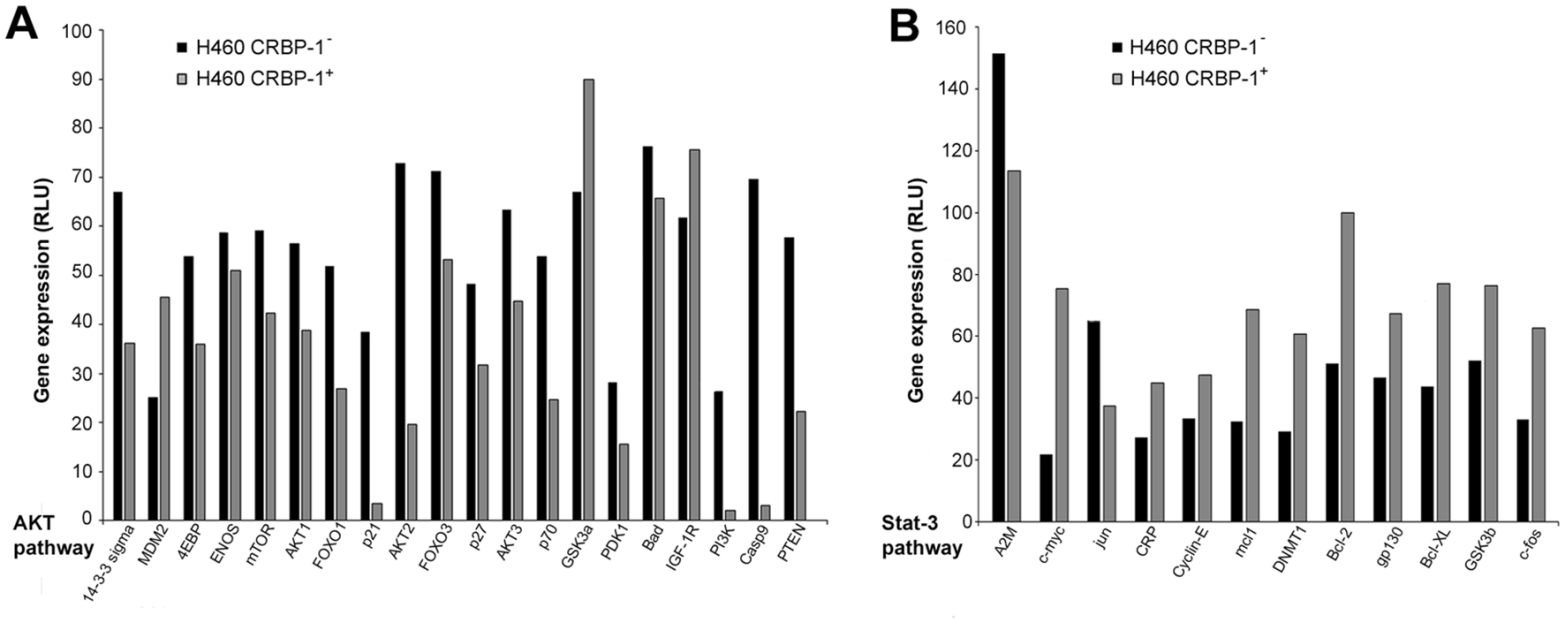
CRBP-1 transfection influences transcriptional pathways and differentiation of H460 cells. **a**, **b** Bar graph of gene array showed gene modulation of Akt and Stat-3 pathways in CRBP-1^+^ compared to empty-transfected H460 cells

**Fig. 3:**
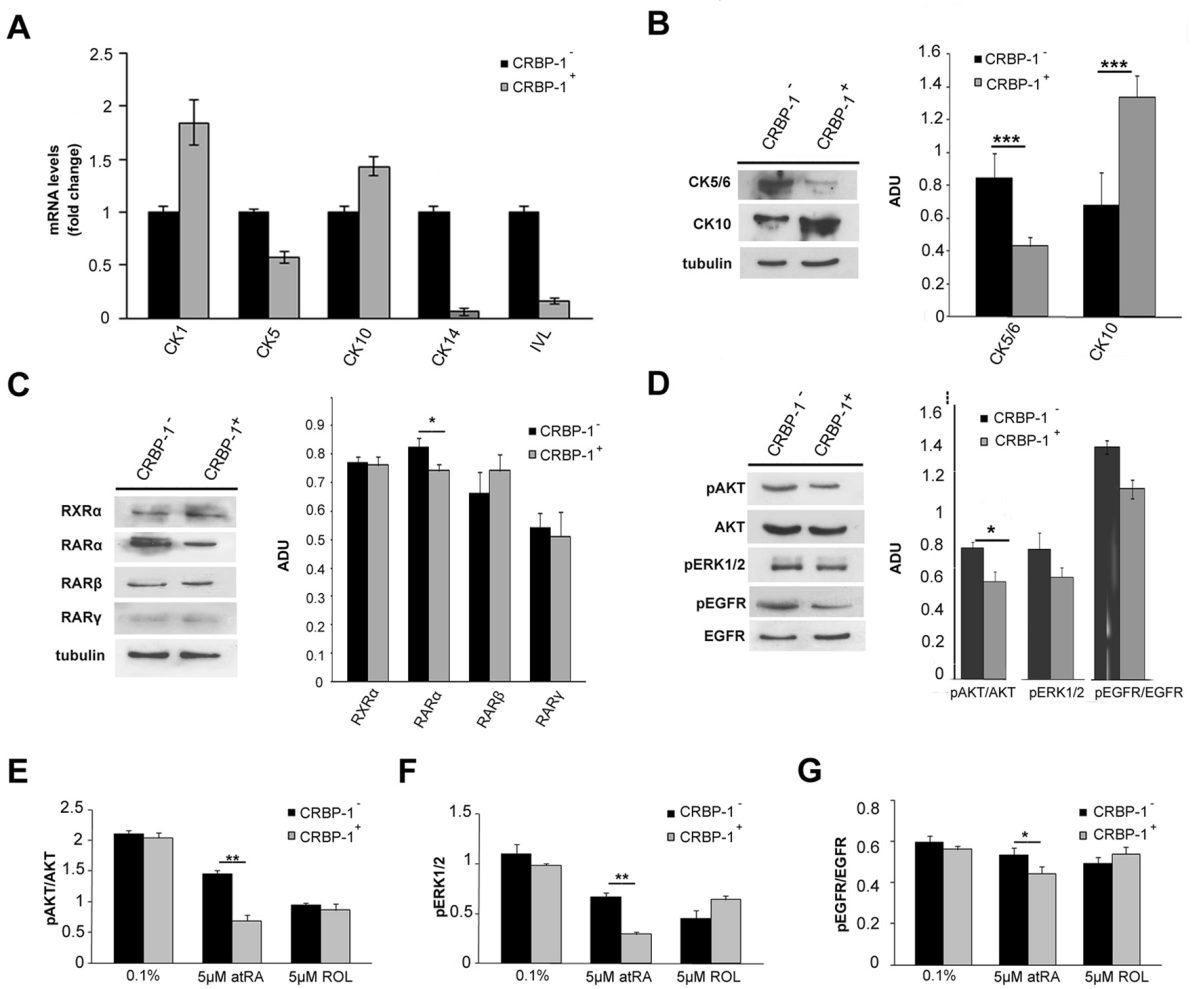
CRBP-1 transfection modulates cytokeratins and Akt-related genes and protein expression in H460 cells.** a** Cytokeratins mRNA levels by real-time PCR; representative blot and bar graph of cytokeratins (**b**), RARs/RXR (**c**), pAKT/AKT, pERK1/2 and pEGFR/EGFR (**d**) protein expression in CRBP-1^+^ compared to empty-tranfected H460 cells maintained with 10% FBS; **e**–**g** Bar graphs of pAKT/AKT, pEGFR/EGFR and pERK1/2 protein expression in CRBP-1^+^ compared to empty-tranfected H460 cells after 48 h of treatment with 5 µM atRA and ROL. *p < 0.05, **p < 0.005 and ***p < 0.001. *RLU* relative light unit, *ADU* arbitrary densitometric units

In order to investigate the CRBP-1-regulated intracellular signalling, gene expression and western blotting analysis were performed. A series of genes involved in AKT and Stat-3-related proliferation pathways resulted modulated in CRBP-1^+^ H460 compared to empty-transfected H460 cells. The down-regulated genes were: 14-3-3-sigma, 4E-BP, Enos, m-Tor, Akt1, Akt2, Akt3, Foxo1, Foxo3, p21, p27, p70, PDK1, Bad, PI3K, PTEN, Casp9, A2M and Jun; while Mdm2, Gsk-3a, IGF-1R, C-myc, CRP, Cyclin-E, MCL1, DNMT1, Bcl-2, GP130, Bcl-xl, GSK-3B, C-fos resulted up-regulated (Fig. 2a, b). Overall, CRBP-1 expression seemed to reduce the expression of proliferative proteins and to increase the expression of differentiation markers. Moreover, we documented the upregulation of cytokeratin-1 and 10 (*p* < 0.02 and *p* < 0.01, respectively) and the down-regulation of cytokeratin-5 (*p* < 0.01) expression in CRBP-1^+^ compared to empty-transfected H460 cells; protein expression for gave similar results (Fig. 3a, b).

In basal condition (10% of FBS), RARα protein expression resulted modulated in CRBP-1^+^ H460 compared to empty-transfected H460 cells, whereas CRBP-1 did not significantly influence RARβ, RARɣ, and RXRα expression (Fig. 3c). As reported in Fig. 3d, we observed the reduction of pAKT expression (*p* < 0.05), but no significant difference in pERK and pEGFR expression in serum-cultured CRBP-1^+^ compared to empty-tranfected H460 cells. Moreover, we showed the downregulation of pAKT (p < 0.05), pERK (p < 0.05) and pEGFR (p < 0.5) expression after 48 h of 5µM *at*RA treatment. Instead, ROL treatment did not influence protein modulation (Fig. 3e–g).

### CRBP-1 expression influences AKT signaling pathway in vivo

**Fig. 4:**
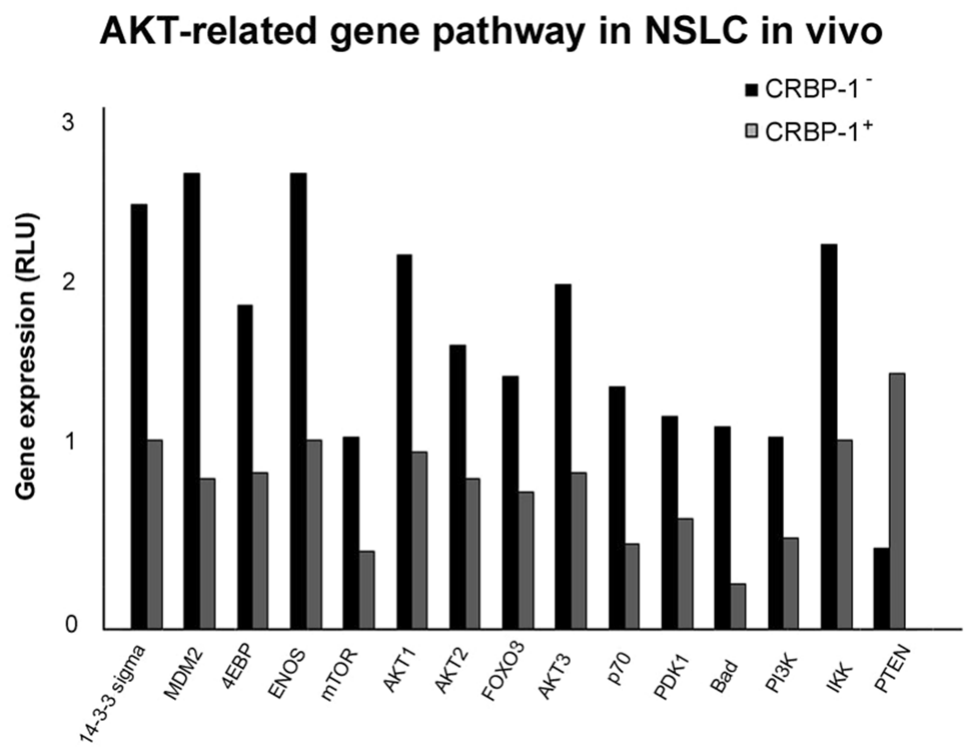
CRBP-1expression associates to modulation of AKT-related genes in NSCLC tissue. Bar graph of gene array showed the modulation of Akt-related gene in CRBP-1 positive compared to CRBP-1 negative NSCLC tissue samples

To further investigate if CRBP-1 positive expression could modulate AKT pathway in vivo, a preliminary gene array was performed. One patient with CRBP-1^+^ tumor and one patient with CRBP-1^−^ tumor expression were analysed. As reported in Fig. 4, genes implicated in AKT pathway resulted down-regulated, whereas only PTEN was up-regulated.

## Discussion

The H460 cell line represents a suitable experimental model of highly proliferative, drug-resistant, metastatic NSCLC cell line [7]. Recently, it has been demonstrated the presence of cancer stem cells in H460 cell line [31] thus explaining at least in part its aggressiveness. It is well documented that chemio and radiotherapy resistance is mainly due to so-called cancer stem cells selection, epigenetic mechanisms silencing physiological pathway (for example hypo or hypermethilation of genes) and finally the acquisition of mutations [32]. In fact, also with the promising tyrosine kinase inhibitors or the immunotherapy patients develop inevitably progression [28, 33, 34]. *At*RA is well known to have a dramatic effect on M3 subtype of acute myeloid leukemia [34]. However, in most other cancers this effect was not observed because of epigenetic silencing of retinoid pathway [35]. Low doses of *at*RA (20 mg/m^2^/day) in combination with chemotherapy showed remarkable activity as demonstrated in a randomized phase II trial of patients with advanced NSCLC [6]. Our results showed a reduced viability and the down-regulation of several genes and proteins involved in proliferative and transcriptional pathways in CRBP-1^+^ compared to empty-transfected H460 cells in both basal condition and after *at*RA treatment. *At*RA is often used in clinical trials to suppress the growth and progression of different cancer types [18, 36]. However, its effectiveness is limited in some cancer, including lung cancer [6, 37, 38]. *A*tRA is an active metabolite of Vitamin A that regulates diverse cellular functions such differentiation, proliferation and apoptosis by binding with RAR/RXR receptors [8]. RARβ is a tumor suppressor gene whose expression is significantly decreased in human cancers and increased with *at*RA treatment [39]. The loss of RARβ mRNA expression has been observed in many lung cancer cells lines and its expression is influenced from intracellular concentration of retinoids, mediated by CRBP-1 [20]. Besides trafficking, CRBP-1 loss is responsible for intracellular retinoid deficiency, since CRBP-1 is required for retinol bioconversion [40–42]. Epidemiological studies suggested that the addition of *at*RA or synthetic retinoids to human cancer cell lines or human tumor xenografts in nude mice results in a cell growth arrest, apoptosis or differentiation [43]. Expression of CRBP-1 may help to switch between a proliferative and differentiative phenotype in response to oncogenetic stimuli [44]. It has been reported that in human mammary tumor cells the reintroduction of CRBP-1 reduces tumorigenicity in athymic mice [45]. Moreover, the inhibition of PI3K/Akt pathway was involved in the antitumor effect of CRBP-1 mediated from p85 regulatory and p110 catalytic subunit heterodimerization [46]. It is possible to hypothesize that re-expression of PI3K/Akt signaling mediates CRBP-1 loss-induced cancer progression and RARβ down-regulation in a transcription-independent mechanism of action of *at*RA.

Several in vitro studies showed that *at*RA induces a transcription-independent activation of the PI3K/Akt pathway [18, 24]. For that reason, we transfected H460 lung cancer cell line with CRBP-1 and compared the effect of *at*RA in Akt signalling and RAR expression. We observed that restoration of CRBP-1 expression in H460 cells reduces survival and clonogenicity compared to empty-transfected cells. Moreover, CRBP-1^+^ H460 cells showed a poor ability to form colonies compared to CRBP-1^−^ cells, both in basal condition and after 48 h of *at*RA treatment.

A series of genes involved in Akt and Stat-3 related proliferation pathways were down-regulated in CRBP-1^+^ H460 cells as Akt1, Akt2, Akt3, Foxo3, p21, p27, p70, Casp9, Gsk-3a, IGF-1R, PI3K, C-myc, Cycline-E, whereas, Bcl-2, GP130, Bcl-xl, GSK-3B, C-fos genes were up-regulated. Those findings confirmed the hypothesis that CRBP-1 expression in H460 cells reduces the expression of proliferative and increases that of differentiation markers. As concerning cytokeratin expression, we observed the upregulation of cytokeratin-1 and 10 and the down-regulation in cytokeratin-5 expression in CRBP-1^+^ compared to empty-transfected H460 cells. In basal condition, CRBP-1^+^ H460 cells showed a significant reduction of pAKT protein expression, but no significant difference in pERK and pEGFR expression. A greater and significant downregulation of pAKT, pERK and pEGFR in CRBP-1^+^ compared to empty-transfected H460 cells was documented after 48 h of 5 µM *at*RA treatment, but not after retinol treatment. As possible explanation, it has been reported that genes related to retinoid biosynthesis, transport, degradation and signaling are deregulated in most NSCLC [47], possibly explaining at least in part those contradictory results. In particular, retinol is transformed in retinal through many enzymes, all of which may be inactivated or non-functioning [47]. Finally, we showed that RARα expression was decreased and RARβ increased in CRBP-1^+^ where compared to empty-transfected H460 cells. Therefore, CRBP-1 restoration influenced proliferation and AKT signaling pathway in H460 cell line also in the presence of *at*RA treatment. Both *at*RA and retinol were dissolved in DMSO, which acts as a histone deacetylase inhibitor. The DMSO action could be a limitation in our study. Nevertheless, being used for the dissolution of both drugs, the differences reported cannot be due to the presence of DMSO. Finally, further studies are needed to define the *at*RA role in CRBP-1^+^ H460 cell line and to clarify if those CRBP-1-related pathways involved in NSCLC carcinogenesis may be modulated, with a potential beneficial opportunity for a more personalized chemotherapeutic regimens employing adjuvant retinoid therapy.

## Data Availability

The datasets used and/or analyzed during the current study are available from the corresponding author on reasonable request.
